# Evaluation of the Intensive Acute Flaccid Paralysis Surveillance System in Ghana: Post the Switch from tOPV to bOPV

**DOI:** 10.3390/tropicalmed9110271

**Published:** 2024-11-08

**Authors:** Evangeline Obodai, Jessica Dufie Boakye, Nana Afia Asante Ntim, Gayheart Deladem Agbotse, Comfort Nuamah Antwi, Ewurabena Oduma Duker, Sharon Ansong Bimpong, Deborah Odame, Patience Lartekai Adams, Josephine Nayan, Jude Yayra Mensah, Angelina Evelyn Dickson, Keren Attiku, Isaac Baffoe-Nyarko, Dennis Laryea, John Kofi Odoom

**Affiliations:** 1 Virology Department, Noguchi Memorial Institute for Medical Research, College of Health Sciences, University of Ghana, Legon, Accra P.O. Box LG 581, Ghana; eobodai@noguchi.ug.edu.gh (E.O.); nntim@noguchi.ug.edu.gh (N.A.A.N.); cantwi@noguchi.ug.edu.gh (C.N.A.); pladams@noguchi.ug.edu.gh (P.L.A.); jonayan@noguchi.ug.edu.gh (J.N.); jymensah@noguchi.ug.edu.gh (J.Y.M.);; 2 Disease Surveillance Department, Ghana Health Service, Ministry of Health, Accra P.O. Box M 44, Ghana

**Keywords:** acute flaccid paralysis (AFP) surveillance, World Health Organization (WHO) indicators, Regional Reference Polio Laboratory, Ghana

## Abstract

The Global Polio Eradication Initiative was adopted by Ghana in 1996, and through robust AFP surveillance was able to interrupt the circulation of wild poliovirus in 2008. However, the country suffered vaccine-derived poliovirus type 2 outbreaks in 2019 and 2022. We conducted a retrospective analysis of all AFP surveillance data received by the polio program in Ghana from 2018 to 2022. An analysis of the WHO performance indicators for evaluating a surveillance system was conducted using Epi Info 3.5.4 and Microsoft Excel. Of the 4832 cases investigated, 56.3% were males, 71.1% comprised children aged 5 years and below, and more than half (65.2%) had received a maximum of three doses of OPV. Over 77% (3028) had a fever at the onset of paralysis, and 67.8% had paralysis progression within 3 days. The non-polio AFP rate of ≥2 and the stool adequacy rate exceeded the target of ≥80% in nearly every study year. The proportion of non-polio enteroviruses isolated surpassed the target of ≥10% in all years except 2018. The AFP surveillance system in Ghana is sensitive and representative. Though the surveillance became more intensive and proactive during the outbreak, the system needs to focus on improving the completeness of the data as well as the timeliness of the arrival of stool specimens within 3 days of collection.

## 1. Introduction

Polio was a known disease affecting people for millennia, but one of the earliest scientific description of this disease was made in 1840 by Jacob Heine [1]. After its recognition in 1840, poliomyelitis was found to infect approximately 350,000 children globally each year. It is a paralytic illness, and 1 in 200 infections leads to irreversible paralysis known as acute flaccid paralysis (AFP) [2,3]. The World Health Assembly (WHA) in 1988 established a resolution for the eradication of polio worldwide, marking the launch of the Global Polio Eradication Initiative (GPEI), which became one of the most extensive public health interventions in the history of disease eradication [4]. In Ghana, the GPEI program was officially recognized in 1996 when African Ministers of Health in 1995 adopted the WHA resolution asking member states to implement the GPEI. This allowed Ghana to increase routine polio immunization, carry out supplementary immunization activities (SIAs), and establish active AFP surveillance for poliovirus with full laboratory support [5].

A sensitive AFP surveillance system remains a central tool for the polio eradication initiative. Hospital surveillance and community-based detection techniques (wastewater surveillance) are used to report and investigate any AFP cases that are discovered [6]. WHO recommends that a person of any age with poliomyelitis suspected by a clinician or any child below the age of 15 who presents with abrupt onset muscle weakness affecting the limbs should be classified as an AFP case for investigation. This broad definition of AFP is also able to capture other non-polio enteroviruses, Guillain–Barré syndrome, transverse myelitis, encephalitis, and traumatic neuritis since they are implicated in AFP [7]. AFP performance indicators and targets have been established by the WHO for accredited laboratories within the Global Polio Laboratory Network (GPLN). The performance indicators track the effectiveness of the AFP surveillance at national and subnational levels, identify gaps where poliovirus (PV) transmission could occur undetected, provide evidence of PV circulation being interrupted, and enable timely detection of outbreaks [6].

With the implementation of the GPEI program in Ghana, the last case of indigenous wild-type poliovirus (WPV) was recorded in 1999 in the northern part of the country [8]. However, further outbreaks of WPV occurred in 2003 and 2008, with viruses genetically linked to those circulating in neighboring countries [4]. After investigating and responding to the epidemic, following WHO guidelines for effective AFP surveillance and continuous Supplemental Immunization Activities (SIAs) with oral polio vaccine (OPV), WPV transmission was interrupted. After many years in which the country did not suffer any more WPV outbreaks, Ghana was declared a polio-free country [5,9].

The AFP surveillance was last evaluated in 2014 [5]; since then, the country switched from the use of trivalent oral polio vaccine (tOPV) for routine immunization to bivalent oral polio vaccine (bOPV) in April 2016. In addition, the country introduced the inactivated polio vaccine (IPV) into the routine immunization schedule in 2018. Between 2019 and 2022, Ghana experienced two cVDPV2 outbreaks. The first episode was detected in sewage samples collected from two environmental surveillance sites (Koblimahgu in the Tamale Metro and Agbogbloshie in Accra Metro) in the year 2019 [8]. During this same year, the AFP surveillance revealed the circulation of cVDPV-2 in children in Ghanaian communities, which lasted until 2020 but was interrupted with intensive and proactive surveillance and three rounds of the monovalent oral polio vaccine type 2 (mOPV2). This was followed by another outbreak in 2022 that was also interrupted within the same year with the novel oral polio vaccine type 2 (nOPV2). The cVDPV2 strains detected from the two outbreaks were found to be importations from Nigeria and belonged to the emergence groups NIE-JIS-1 and NIE-ZAS-1, respectively, and appear to carry the greatest risk of cVDPV2 emergence, mimicking AFP cases of the WPV [10].

The need to continually evaluate the AFP surveillance system is key to ensuring that it is robust enough to guide public health responses in the complete eradication of WPV worldwide and the ending of cVDPV outbreaks. This study analyzed the epidemiological distribution of AFP cases across Ghana over a 5-year period (2018–2022) and evaluated the more intensive and proactive AFP surveillance system during the outbreak, using standard performance indicators recommended by the WHO to identify areas that require improvement as a way of maintaining Ghana’s polio-free status [6].

## 2. Materials and Methods

### 2.1. The Study Area and Design

Ghana is a West African country that, until 2018, consisted of 10 regions but now has 6 more, summing up to sixteen regions. The projected population, based on the growth rate of 2.1% from the 2021 Population and Housing Census, was 30,832,019. Children below the age of 15 constitute about 36% of the nation’s population.

A retrospective study was conducted by analyzing data collected from January 2018 through December 2022 by the Disease Surveillance Department of the Ghana Health Service (GHS) and the WHO-accredited Regional Reference Polio Laboratory (RRPL) in Accra, Ghana. All reported AFP cases from the sixteen regions of Ghana were included in the study.

### 2.2. The AFP Surveillance System in Ghana

The Expanded Program on Immunization was developed in Ghana in 1974 and became operational in all regions by 1985. Routine polio immunization was carried out using tOPV before it was switched to bOPV and IPV in 2016 and 2018, respectively. The routine polio immunization program for children includes four doses of live attenuated bivalent oral polio vaccine (bOPV). Children receive their first vaccination (OPV0) at birth. The remaining three doses (OPV1, OPV2, and OPV3) are given at 4 weeks, 10 weeks, and 14 weeks after birth, respectively. During OPV3, IPV is also administered. The Disease Surveillance Department (DSD) of the Ministry of Health in Ghana’s case definition for AFP includes any child aged below 15 years who develops sudden weakness in the limbs. According to WHO, all AFP cases should be reported immediately and investigated within 48 h and that two stool specimens should be collected 24–48 h apart and within 14 days of the onset of paralysis. A country’s surveillance system is expected to detect at least two cases of AFP per 100,000 children under 15 years of age, and it is recommended that a minimum of 80%of AFP cases should have adequate stool specimens [11]. When a child meets the AFP case definition and reports to a health facility, the DSD is notified, and an elaborate investigation is carried out by the health practitioners to determine the cause. This commences with filling out a case investigation form containing sections for demographics, clinical history, immunization history, and details needed for laboratory investigations. The investigation continues with the collection of adequate stools (two stool specimens, 24 to 48 h apart, within 14 days of the onset of paralysis). Stool specimens and their corresponding forms are transported to the WHO-accredited Regional Reference Polio Laboratory (RRPL) at the Noguchi Memorial Institute for Research to be tested.

The polio laboratory in Ghana was designated as the first WHO-accredited RRPL in the African region in 1992 and became the Centre of Excellence for the organization of training programs on the laboratory diagnosis of polio, measles, and yellow fever to build the Polio Laboratory Network in the African region from 1992 to 2000. The WHO-accredited RRPL in Ghana follows standardized protocols to (i) process and treat stool specimens to isolate the virus by cell culture; (ii) differentiate between the poliovirus serotypes 1–3, WPVs, Sabin-like polioviruses, and the vaccine-derived polioviruses by intra-typic differentiation; (iii) sequence the VP1 region of all wild and VDPV isolates to determine their origin; and (iv) send all WPVs and VDPVs to a WHO-specialized Polio Laboratory for closest match and final classification and nOPV2 isolates for whole genome sequencing. The polio laboratory also isolates and characterizes non-polio enteroviruses to identify those implicated in causing paralysis in humans, and shares results with the DSD. The three standard laboratory timeliness indicators for stool specimen processing are to report ≥80% isolation results within 14 days of receipt, report >80% ITD results within 7 days of receipt of specimens, and ship ≥80% of WPV and suspected VDPV isolates to the sequencing laboratory within 3 days of ITD results. The independent programmatic standard indicator is to report ITD results for ≥80% of isolates within 60 days of the paralysis onset of persons with AFP cases; this indicator considers the entire interval from the onset of paralysis through case notification, and investigation, as well as specimen collection, transport, and testing. In addition to timeliness, the accuracy and quality of laboratory testing are monitored through an annual accreditation program of onsite reviews and proficiency testing [11,12].

### 2.3. Vaccine Coverage and Supplemental Immunization Activities

We counted the number of AFP cases with up to 3 doses of bOPV and IPV1, determined the proportion, and compared it with the national coverage. We also analyzed immunization data collected during cVDPV2 outbreaks to assess the vaccination status and level of immunity, as well as the immunization gaps contributing to the outbreaks.

### 2.4. Outbreak Investigations

The Polio Laboratory reported the first case of AFP due to cVDPV type 2 from a 31-month-old girl from Ando Nyamanu in the Chreponi district of the of the northeast region. Following the report, a team comprising staff from the Ghana Health Service (GHS) and Health Partners visited the Regional Health Directorate and conducted intensive investigations, including desk reviews of available AFP health reports, interviewing relevant stakeholders, reviewing the patients’ health records at the Chereponi hospital and nearby clinics, and conducting active case searches for AFP. Just as the investigations were completed, several other cases were detected from the Savanna, Oti, Ashanti, Bono, and Ahafo regions.

The country has a National Polio Expert Committee (NPEC) that meets quarterly to conduct the final classification of all AFP cases. An AFP case in which two adequate stool specimens are submitted for analysis and no poliovirus is isolated is classified as a non-polio case (and is said to have been discarded). A case in which the stool specimens are deemed inadequate but have no residual paralysis after 60 days of the onset of symptoms is also classified as a non-polio case (discarded). A case that has inadequate stool specimens and has residual paralysis after 60 days of the onset of paralysis, or where the patient is lost to follow-up or dies within 60 days of the onset of symptoms, are classified as compatible with polio or discarded. The WHO has devised a set of performance indicators to ensure that AFP surveillance is properly maintained. We evaluated the quality of the AFP surveillance using the WHO guidelines for minimum performance standards [6,11,12,13].

### 2.5. Data Analysis

All data entry was performed using Epi info software (version 3.5.4) and extracted in Microsoft file formats, which included all key demographic, clinical, vaccination, and polio surveillance variables. Descriptive statistical analysis was conducted using Microsoft Excel (version 2021) spreadsheets for key polio surveillance indicators, and findings were presented using figures, charts, and tables based on the WHO-recommended AFP surveillance performance indicators [6,13,14].

### 2.6. Ethical Considerations

A waiver for ethical approval for the study was obtained from the Institutional Review Board of the Noguchi Memorial Institute for Medical Research. Approval was also granted by the Disease Surveillance Department of the Ghana Health Service. We protected the confidentiality of patients by using codes.

## 3. Results

A total of 4832 AFP cases were investigated during the period of review. Of the samples collected, 56.3% were from males, and 71.1% were from children aged 5 years and below. Children aged 6 to 15 constituted 24.9%, and 1.0% of the cases were above 15 years of age. Regarding immunization status, 65.2% had received a maximum of three doses of bivalent Oral Polio Vaccine (bOPV), 15.8% had received at least four OPV doses, and 90.8% had received IPV1, whilst 19.0% accounted for those with unknown immunization status (Table 1). The national coverage at the time was 99% for both routine immunization and IPV. The clinical history of the study population showed that an average of 77.8% had fever at the onset of paralysis; the proportion with progression of paralysis within 3 days was found to be 67.8%, and 54.3% presented with asymmetric paralysis.

**Figure 1:**
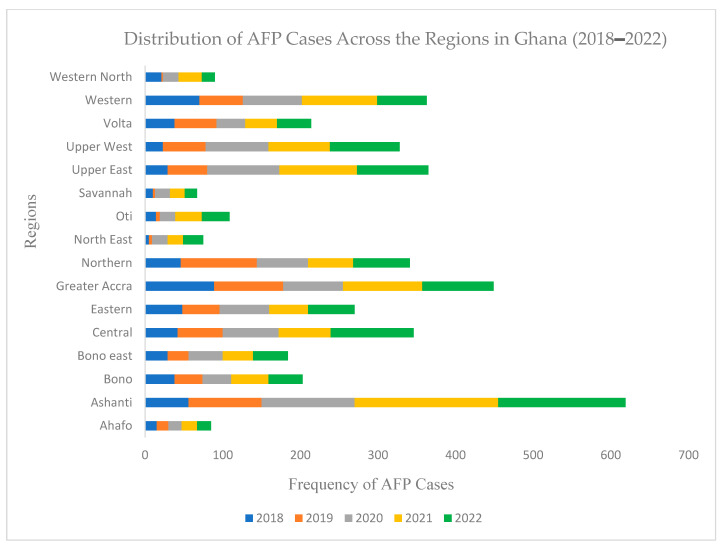
Distribution of AFP cases across the regions in Ghana from 2018 to 2022.

During the five-year study period, all patient samples that met the AFP case definition were received and processed in the lab. Figure 1 shows the distribution of AFP cases across the country, with the Ashanti region recording the highest proportion. All sixteen regions detected AFP cases, with the six newly created regions in 2020 detecting the least, indicating the representativeness and acceptability of the AFP surveillance system across Ghana. Overall, the number of AFP cases detected during the outbreak from 2019 to 2022 for each year was about twice that of 2018 when there was no outbreak.

**Figure 2:**
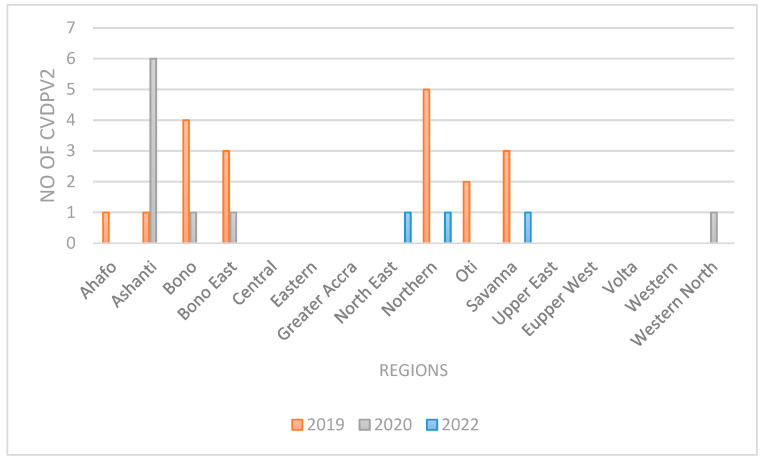
Number of cVDPV 2 cases that were detected during the outbreak, 2019–2022. The outbreaks occurred in three of the five-year study periods.

The cVDPV2 outbreak continued until 2020, before it was interrupted. There was no outbreak in 2021, but the country suffered another cVDPV2 outbreak in 2022. Figure 2 shows the distribution of cVDPV2 over the period in the regions. In 2019, when the outbreak started, the virus was detected in seven regions, with the highest in the northern region. In 2020, cVDPV2 was detected in four regions, while in 2022, the virus was found in three regions.

Table 2 shows the polio immunization coverage during the response activities. Prior to the cVDPV2 outbreak, Ghana had conducted a Sub-National Immunization Day (SNID) in 2018 within three regions. During the 2019–2020 outbreak, several Sub-Immunization Activities (SIA) were conducted using the monovalent oral polio type 2 vaccine (mOPV2). Due to the instability of the mOPV2, SIAs were conducted in phases (a few districts at a time) depending on where the virus was circulating at a particular time. In 2021, the WHO released a new, more stable oral polio vaccine, the novel oral polio vaccine type 2 (nOPV2), for the polio campaign. Two rounds of National Immunization Days (NIDs) were conducted with the nOPV2 to interrupt the circulation of cVDPV2 in 2022.

Table 3 shows the evolution of WHO-established AFP performance indicators in Ghana during the study period and includes the performance target for each indicator. The two most important indicators for the sensitivity of the system are non-polio AFP rate (NPAFP) and stool adequacy. With respect to the former, the system greatly exceeded the target of 2 per 100,000 children under 15 years of age throughout the study period. The stool adequacy rate, which is measured by the number and date of collection of stool specimens, and the condition of specimens upon arrival in the RRPL lab, surpassed the set threshold in all years except in 2020, where it was around 79%. Over 90% of the specimens collected reached the laboratory in good condition. The timeliness of stool specimens reaching the laboratory within 3 days of collection was not met except in 2019. The laboratory indicators, including results turnaround time and non-polio isolation rate, were met. Over the 5-year study period, the virus isolation result within 14 days of receipt in the laboratory was met, and the non-polio enterovirus isolation rate exceeded 10% for the entire period. As shown in Table 3, a total of 31 AFP cases due to cVDPV2 were detected in the country during the first outbreak, with 19 occurring in 2019. During the second outbreak, however, only three AFP polio cases due to cVDPV2 were detected, bringing the total AFP polio cases due to cVDPV2 detection over the 5 years to 34 cases, and that due to AFP contact without polio but with the isolation of cVDPV2 to 33.

**Figure 3:**
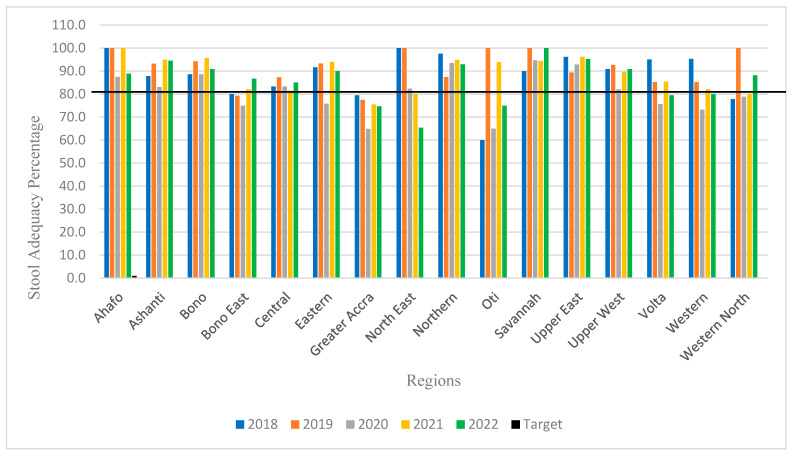
Stool adequacy rates per year, per region, from 2018 to 2022.

As mentioned earlier, the stool adequacy over the period exceeded the WHO target of 80%. In terms of region, however, only sevenix of the sixteen regions, including Ahafo, Ashanti, Bono, Northern, Savanna, Upper East and Upper West, met the 80% for the entire 5 year period. The Greater Accra region did not meet the target for the entire 5 year period. Greater Accra’s best performance was in 2018, with 79.5%, and its worst was in 2020, with 64.9%, The remaining nine regions did not meet the 80% target in 1, or 2, or 3 years over the entire period of 5 years, as shown in Figure 3.

## 4. Discussion

We evaluated the AFP surveillance indicators in Ghana from 2018 to 2022 after the switch from tOPV to bOPV, the introduction of IPV, and during the period when cVDPV2 circulated in the country. During the period from 2019 to 2022, the number of AFP cases doubled compared to 2018, when there were no outbreaks. This could be due to the intensive and proactive surveillance introduced, which allowed staff to actively look for AFP cases instead of the normal passive method of surveillance. The demographic and clinical variables showed that more than half of the AFP cases were males, and more than half were less than 5 years old and had a fever at the onset of paralysis, with progression ≤ 3 days. Children who had received three or more doses of bOPV through routine or SIAs also comprised more than half. The study found that the two most important AFP surveillance indicators (NP-AFP rate and stool adequacy) exceeded the WHO target; however, some regional differences were also seen in the stool adequacy performance. A sixty-day follow-up was also conducted for more than half of the AFP cases. These findings highlight the strengths and gaps in the surveillance system and recommend areas for improvement for the country to sustain its polio-free status.

Our findings revealed proper documentation of demographics, clinical history, and immunization profile, which are key to improving surveillance performance indicators. In terms of data completeness and quality, the routine bOPV2 coverage consistently declined from 2018 to 2022. This observation could be due to the impact of the SARS-CoV-2 pandemic on vaccine preventable diseases. This is similar to our previous findings [8], where we hypothesized that parents were unable to recall the exact vaccination details of their wards. A study conducted in South Africa also found weaknesses in the documentation of laboratory results in their AFP database, which negatively impacted data quality [15].

Studies have shown that age and sex are risk factors associated with AFP distribution [7]. We found that the majority (70%) of the AFP cases were <5 years old, the age group where children are mostly affected by poliomyelitis, and prevalence was higher in males than females [16]. This is an indication that the surveillance system could detect cVDPV and wild poliovirus if present. This finding is not different from our previous study [8], in Nigeria [17] and Ethiopia [18]. The average proportion of AFP cases with fever at the onset of paralysis was found to be 77.8%, with 54.3% having asymmetric paralysis, and 67.8% having progression to paralysis with 3 days. Sensitive AFP surveillance is crucial for detecting poliovirus transmission and relies on rapid case identification, notification, investigation, specimen transport, and laboratory testing. The primary indicator for assessing the sensitivity of an AFP surveillance system is the non-polio AFP, which assesses the system’s capacity to detect WPV in the event of re-importation into the country [15,19]. The WHO NP-AFP rate target of ≥2/100,000 was exceeded throughout the study period, steadily increasing from 2018 to 2022. This represents an improvement over an earlier study in Ghana that showed that the non-polio-AFP target was only achieved in three of the five-year study periods. The findings were also similar to previous studies [4,20,21], with higher average annualized rates reported in Nigeria [17]. The second most important indicator, the stool adequacy rate, on average, exceeded the WHO target, which also speaks to the sensitivity of the system. However, when the results are segregated into regions, nine of the sixteen regions were unable to attain the 80% target throughout the five years, including Greater Accra, the capital region of Ghana. This could be attributed to the late collection of stools as a result of knowledge and logistics gaps. It could also be due to inadequate logistics for sample packaging and transportation or the inability to maintain the reverse cold chain, leading to stools arriving in the laboratory with temperatures above 10 °C [22]. Samples of this nature tend to be classified as polio-compatible by the Polio Expert Committee, which negatively impacts the surveillance program. Our analysis also showed that the recommended ≥80% of stool samples arrived in the laboratory ≤3 days of collection was only met in 2019. The investigations found that samples from the districts were batched at the regional level before they were sent to the polio laboratory. This has serious consequences for virus isolation, depending on the storage temperature and the reverse cold chain for transporting samples to the lab. The results of our analysis also showed that, even though timeliness was not met, over 90% of the samples reached the polio laboratory in good condition. This is only possible when the regions repackage the samples, change the ice packs, and transport them to the polio laboratory. The non-polio enterovirus (NPEV) rate, as a measure of sensitivity, analyzes how well the AFP surveillance system can maintain the reverse cold chain, and it evaluates how well laboratories execute routine enterovirus isolation [17]. Apart from 2018, all years across the evaluation period outperformed the minimum ≥ 10% NPEV target. This is consistent with a study conducted in Sokoto State, Nigeria, where the NPEV rate was met throughout the study period, except one particular year with a rate of 9.5% [17].

Our findings also indicated that two cVDPV2 outbreaks occurred during the period. This was the first time the country experienced a cVDPV2 outbreak after the implementation of AFP surveillance in 1996. The first outbreak lasted from 2019 to 2020, while the second occurred in 2022 and was interrupted within six months. A total of 114 cVDPV2 cases were confirmed over the period, with 46 from the environment, 34 from AFP cases, and 34 from AFP contacts. Investigations indicated that the first cVDPV2, a sample collected from a 9-month-old girl on 25 August 2019 in Chereponi District, was genetically linked to the type 2 circulating vaccine-derived poliovirus NIE-KWS-KSB-18-006 HC29, which appeared and was first detected in Nigeria in 2018. The ability of the surveillance system to detect cVDPV2 from humans confirms its quality and sensitivity and needs to be strengthened, especially in districts where surveillance indicators are weak. Our findings further indicated that the SIAs were carried out in phases with the monovalent oral polio vaccine type 2 (mOPV2) during the first outbreak due to the inherent ability of the mOPV2 to transmit to others. Notwithstanding this, the virus spread to many regions, each time, “chasing” the virus with the vaccine. Although routine immunization and SIAs continued, the impact of COVID-19 slowed down activities, allowing transmission to continue for one year before it was interrupted. During the second outbreak, however, two rounds of NIDs were conducted with nOPV2, thereby interrupting transmission earlier. The quality of the country’s immunization program appeared good, but areas like coverage, cold chain, and transportation need to be strengthened.

The limitations observed in this study include incomplete documentation of some demographic information on the age of the patients, clinical information on the records of immunization, and the quality of coverage information. Hence, we recommend that surveillance officers conducting case investigations must source all relevant information to be captured in the database to enhance data analysis.

## 5. Conclusions

In conclusion, the evaluation provided evidence of a strong and robust AFP surveillance system that is sensitive, capable of early detection and quick response, but also demonstrates the need to strengthen the system in terms of data collection and timeliness of sending samples to the laboratory. Although the WHO targets for the NP-AFP rate and stool adequacy were met at the national level, strengthening at the regional level is important, considering the ongoing PV transmission in the subregion. Routine immunization should also be strengthened to cover all children, ensuring effective protection to avoid silent transmission of poliovirus going unnoticed. 

## Figures and Tables

**Table 1:** Demographic characteristics of Acute Flaccid Paralysis cases reported in Ghana, 2018–2022.

Characteristic	Frequency (*n* = 4832)					
	2018 (%)	2019 (%)	2020 (%)	2021 (%)	2022 (%)	Average
Sex						
Male	55.3	57.9	58.2	54.8	55.1	56.3
Female	44.7	42.1	41.8	45.2	44.9	43.7
Age Group Categories						
≤5 years	72.1	73.9	69.7	70.9	71.3	71.1
6–15 years	25.7	23.0	26.8	24.5	22.1	24.9
˃15 years	1.4	1.1	0.0	1.6	0.8	1.0
Unknown	0.8	2.0	3.5	3.0	5.8	3.0
Polio Immunization Status (bOPV)						
≤3	74.5	69.2	61.9	61.2	63.5	65.2
≥4	4.3	14.0	19.1	19.5	17.4	15.8
Unknown immunization status	21.2	16.8	19.0	19.3	19.1	19.0
IPV1 immunization status	56	99	95	103	101	90.8
Clinical History						
Fever at onset of paralysis (yes) (%)	82.2	82.8	71.6	78.3	74.2	77.8
Paralysis progressed ≤ 3 days (yes) (%)	71.3	70.9	63.5	70.%	63.0	67.8
Asymmetrical paralysis (yes) (%)	50.4	58.3	54.1	56.2	52.6	54.3

**Table 2:** Polio immunization activities during the cVDPV2 outbreak, 2019–2022.

Date	Phase	NID/SNID	No of Districts (Region)	NID/SNID Coverage	Routine Immu with OPV3 *	Routine Immu with IPV **
September–November 2018	I	SNID	73 (3)		100	56
December 2019–February 2020	II	SNID	38	757,126 (100.4%)	99	99
R1 March 2020	III	SNID	179 (8)	799,680 (106.1%)	92	93
R2 10–13 September 2020		SNID		4,328,624 (94.7%)		
R3 8–11 October 2020		SNID		4,674,448 (102.3%)		
1–4 September 2022	Round I	NID	275 (16)	7,653,922 (111.2%)	90	101
6–9 October 2022	Round II	NID	275 (16)			

* Shows routine OPV3 coverage at the time of the campaign. ** Shows routine IPV coverage at the time of the campaign.

**Table 3:** AFP performance indicators for Ghana, 2018–2022.

Performance Indicators	Target	Country’s Performance				
		2018	2019	2020	2021	2022
Number of cases reported annually	150	556	940	1329	978	1029
Annualized non-polio AFP rate/100,000 < 15 yrs population	≥2	3.6	4.7	5.3	6.1	6.0
Proportion of stool specimens from which non-polio enterovirus was isolated	≥10%	9.0	10.7	14.5	11.8	15.2
Proportion of Specimens collected within 14 days of the onset of paralysis	≥80%	89.0	88.6	81.4	89.8	87.4
Proportion of specimens that arrived at a WHO-accredited laboratory < 3 days of collection	≥80%	76.1	80.2	66.2	67.2	57.3
Proportion of stool specimens with results sent from the laboratory < 14 days of receipt by the laboratory	≥80%	80.8	86.2	86.6	93.6	84.2
Proportion of AFP cases with two adequate stool specimens	≥80%	87.9	86.9	79.2	88.0	86.1
Proportion of stool specimens arriving at the laboratory in good condition	≥80%	94.3	99.0	99.3	99.2	99.1
* Number of cVDPV2 isolated from AFP polio cases	N/A	0	19	12	0	3
** Number of cVDPV2 isolated from AFP contacts	N/A	0	21	10	0	3

* number of cVDPV2 isolated from AFP polio cases from 2018 to 2022. ** number of cVDPV2 isolated from AFP contacts over the outbreak period of 2018 to 2022.

## Data Availability

The original contributions presented in the study are included in the article; further inquiries can be directed to the corresponding author.
